# Body Mass Index in Human Gait for Building Risk Assessment Using Graph Theory

**DOI:** 10.3390/s20102899

**Published:** 2020-05-20

**Authors:** Washington Velásquez, Manuel S. Alvarez-Alvarado, Andres Munoz-Arcentales, Sonsoles López-Pernas, Joaquín Salvachúa

**Affiliations:** 1 Escuela Superior Politécnica del Litoral, Campus Gustavo Galindo Km 30.5 Vía Perimetral, P.O. Box 09-01-5863, Guayaquil EC090112, Ecuador; manuel.alvarez.alvarado@ieee.org (M.S.A.-A.); joseandres.munoz@upm.es (A.M.-A.); 2 Departamento de Ingeniería de Sistemas Telemáticos, ETSI Telecomunicación, Universidad Politécnica de Madrid, 28040 Madrid, Spain; sonsoles.lopez.pernas@upm.es (S.L.-P.); joaquin.salvachua@upm.es (J.S.)

**Keywords:** body mass index, breadth-first search, evacuation routes, human gait, Monte Carlo simulation

## Abstract

This article presents a comprehensive study of human physiology to determine the impact of body mass index (BMI) on human gait. The approach followed in this study consists of a mathematical model based on the centre of mass of the human body, the inertia of a person in motion and the human gait speed. Moreover, the study includes the representation of a building using graph theory and emulates the presence of a person inside the building when an emergency takes place. The optimal evacuation route is obtained using the breadth-first search (BFS) algorithm, and the evacuation time prediction is calculated using a Gaussian process model. Then, the risk of the building is quantified by using a non-sequential Monte Carlo simulation. The results open up a new horizon for developing a more realistic model for the assessment of civil safety.

## 1. Introduction

According to the World Health Organization (WHO), the number of individuals who are overweight worldwide has tripled since 1975 (http://www.who.int/es/news-room/fact-sheets/detail/obesity-and-overweight). In 2016, more than 1900 million adults (18 or older), 340 million children between 5 and 19 and 41 million children under 5 were overweight or obese [1]. The Body Mass Index (BMI) is a reliable indicator of overweight, and it is commonly used to identify if a person lies within a weight category that can lead to health problems [2]. For instance, a person is considered to be obese if his or her BMI is greater than 30. There are a number of studies in the literature in which the BMI is calculated on the basis of weight and height [3,4,5,6,7]. Nevertheless, the Center for Disease Control and Prevention (CDC) states that “factors such as age, sex, ethnicity, and muscle mass can influence the relationship between BMI and body fat. Also, BMI doesn’t distinguish between excess fat, muscle, or bone mass, nor does it provide any indication of the distribution of fat among individuals” [8,9]. Therefore, it is relevant to consider these factors to avoid inaccuracies in BMI calculation.

The new trends in Information and Communication Technologies (ICT) have fostered the development of countless applications in several fields, such as telemedicine and e-health. In most applications, the BMI is used to indicate certain ways in which the physical condition of an individual can be improved [10]. For instance, some Xiaomi products (My Band 2, 3 and My Body Composition Scale) [11] allow customers to keep track of daily activities and, through a mobile application, consult several health indicators such as BMI, body fat, bone mass and muscle mass in a reliable and safe way. Although these applications help citizens become aware of their BMI, there are several other factors influencing this measure such as eating habits [12], lack of daily exercise [13] and stress [14], that affect the daily routine of human beings, especially in the workplace. Taking into account these factors, Mattila et al. [15] presented a concept for maintaining ICT-assisted health in a company, designed to address various risks in occupational safety that allow better management of employee health. Furthermore, Akhtar et al. [16] presented a mixed-methods approach, including user experience and a perception survey through telemedicine services. Both studies focus on monitoring a person’s health by providing the necessary information for a healthy life. Conversely, this study aims to make use of the BMI provided by these applications in order to relate it to the possibility of evacuating a building when an unforeseen event happens.

The main contribution of this article is an innovative model that allows assessing building risk using evacuation times. The evacuation times are calculated on the basis of the nearest emergency exit to each person in the building. The model considers two main algorithms: (1) Breadth-First Search (BFS (https://www.geeksforgeeks.org/breadth-first-search-or-bfs-for-a-graph/) for determining the optimal evacuation route, and (2) Non-Sequential Monte Carlo simulation for determining the risk index of the building. The building employed for validating the model is based on the laboratory of the Institute of Applied Information Technology, which is subject to an earthquake condition. The model incorporates the real size of the different rooms and exit pathways of the building. In order to get realistic results, people with different physiologies are simulated. This model is based on previous research in which the relationships between BMI at different speeds and human gait [17], and between BMI and human walking on different types of surface (concrete, wood and ceramic) are calculated [18]. Moreover, the centre of mass, weight and height of a person are also taken into consideration in this model.

The rest of the article is structured as follows. Section 2 presents related work in the literature. Section 3 describes the mathematical model of human gait expressed as a function of BMI. Section 4 describes the simulation environment including the building’s representation using graphs. Section 5 presents the case study in this analysis. Section 6 presents the test results and an analysis of the impact of BMI on human gait. Finally, a discussion about this research and future work that has been contemplated before the implementation of a reactive emergency system is presented in Section 7.

## 2. Related Work

The existing literature presents different approaches to the study of human gait. For instance, Ackermann et al. [19] presented a model based on walking optimisation using minimal energy to predict how a patient’s gait adapts to mechanical interventions such as prosthetics or surgery, concluding that minimising fatigue may be one of the optimisation principles governing human pace. Likewise, Dorn et al. [20] formulated a predictive simulation framework based on energy minimisation, which they use to simulate a regular walk, with a variety of slopes and loads transported. This simulation is muscle-driven and includes controllers based on muscle force and stretch reflexes. More comprehensive research is presented by Sun and Sakai in [21], in which human motion is predicted using a model based on measurement sensors. Their study focuses on the various angles that legs form when walking at different speeds. Similarly, Balazia and Plataniotis [22] propose a method for gait recognition using image classifiers that can be applied to a street-level video surveillance environment. Following a different approach, Rajagopal et al. [23] presented an open source 3-D musculoskeletal model with hi-fi representations of the lower limb musculature of young individuals that can be used to generate accurate gait simulations. The results suggest that the model they present is suitable for creating adequate muscle simulations for a healthy gait. Although there is a considerable number of studies on human gait, there is a dearth of literature regarding the influence of human gait on public safety, and simulations regarding natural hazards are not explicitly given. For instance, the study conducted by Wolf, Babaee and Rigoll [24] presents a deep convolutional neural network for gait recognition in multiple views. Although this approach evaluates three different datasets (clothing variations, gait speeds and angle of vision), allowing to have comparable performance from different perspectives, it does not study how to improve occupational safety or natural disasters.

When studying evacuation route modelling schemes, different approaches have been followed. For instance, in [25], an evacuation model for low-level fires is proposed. Such model considers people’s motion as if they were in a “fluid”, in order to obtain the maximum evacuation time. It describes different mathematical models for each surface (room, stairs and lobby), in which people are supposed to travel in a fixed direction and angle until they reach the nearest exit. This model has an interesting peculiarity: if the room has more than one exit, it is divided into equal sections (smaller rooms with only one exit). Moreover, if there are obstacles inside the room, the model considers that the person moves in the shape of an “L” in order to reach the nearest exit. Similarly, in [26], an approach based on the fire escape stairs of a high-rise building is presented, in which the scheme of automaton cells are incorporated to human fatigue in the movement of a person to quantify the impact on the evacuation time. The design of the automaton cells is based on cell separation per room, i.e., a different cell for each room, staircase and hall. When a user arrives at the exit of one surface, he/she is automatically transported to the next automaton cell. This process is repeated until the exit is reached. However, in this study, the verification of fatigue was empirically obtained using a real-world scenario, and no mathematical model was defined. In [27], an evacuation model is presented including a study that regards chaotic movement as the main reason for injuries in people in an emergency. This model is aimed at reducing the total evacuation time by leading people to the nearest exit. However, this study is reduced to a simulation of a room with four exits. Furthermore, in [28], the authors present an evacuation model that applies the functions developed by Nelson and McLennan [29] for modelling human movement. Both studies incorporate a new procedure to solve evacuation problems in buildings. Even though these studies consider cinematic parameters (distance, time, etc.) to calculate the evacuation time, they disregard people’s physiology in their analysis (human gait), which brings inaccuracies when applying their proposed models in real scenarios. Likewise, Li et al. [30] present an approach using stairs in a multistorey elementary school, in which the spatial topology of twisted stairs and their relationship with the floors in the event of evacuation are studied. The validation of this study was carried out using simulations of real scenarios for showing qualitative and quantitative consistencies of the simulated results. Finally, Yuan and Tan [31] present a model of the evacuation of a room with obstacles, in which the evacuation analysis is conducted using automaton cells and human behaviour including only the effect of inertia. Overall, there are certain limitations to these studies. For instance, one concern is that they only consider a section of the area of a building, such as the stairs or a single room. Another limitation stems from the fact that the incidence of the body weight of a person in the evacuation is not considered. In addition, there is a lack of mathematical formulation to describe the motion of a person, and they do not acknowledge how well a building is designed to withstand an emergency.

In an effort to fill the gaps in the existing literature, this paper proposes a comprehensive model based on the BMI to determine the evacuation time of a building, taking into account the physical phenomena involved in human motion. The features of the building and the people involved are modelled by using graph theory incorporating a building with different floors, in which each node within the scheme represents a floor (including obstacles, doors, offices) and the relationships between floors represent the stairs (distance between each floor accounted for unit step). The BFS algorithm is used to define the optimal evacuation route, and the Monte Carlo simulation is utilised to indicate the risk index of the building. The proposed approach opens a pathway to bring a more accurate and realistic analysis to this field of study.

## 3. Human Gait Model Based on Bmi

This section describes the mathematical model used to calculate the evacuation time of a building. This model is based on previous research described in [17,18], where a BMI model is designed to recognise incidences on different types of soil.

### 3.1. Centre of Mass

The centre of mass of a human body varies depending on the person’s physiology. Nevertheless, there is evidence [32,33] that it mainly depends on sex and height, *h*. Taking the feet as a reference point, the mathematical formulation to calculate the position of the centre of mass of the human body is as follows.

(1) $$x=kh;k=\left\{\begin{array}{cc} 0.565 & \mathrm{for}\;men\\ 0.550 & \mathrm{for}\;women \end{array}\right.$$

### 3.2. Inertia of the Human Body When Walking

**Figure 1:**
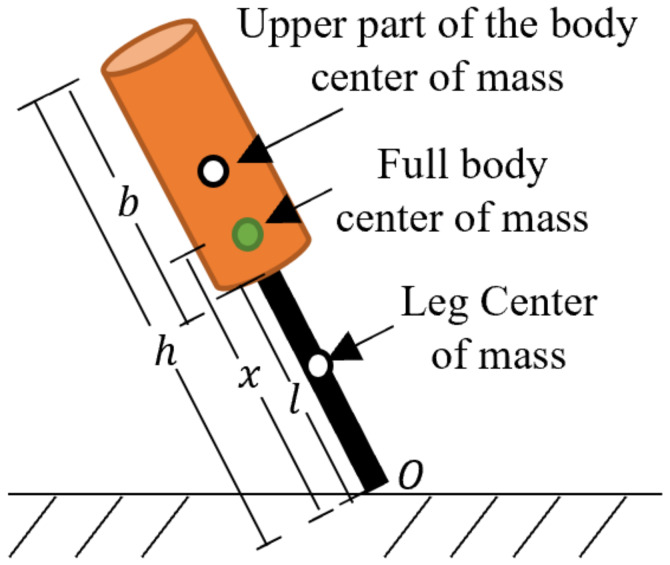
Human body represented as an inverted physical pendulum.

From the physical point of view, an inverted pendulum is a mechanism in which the centre of mass oscillates over a pivot that contacts a solid surface. By analogy, the human gait represents such a mechanism, in which the human body centre of mass moves following repetitive variation (oscillation) with support on the feet (pivot), as presented in Figure 1. This idea is supported by previous investigations, such as in [34], which proposes a new method to simulate human gait motion based on the enhanced version of three-dimensional linear inverted pendulum. The authors of [35] conducted an experimental study with an inverted pendulum apparatus to describe human body motion, resulting suitable for human gait modelling. A more detailed study in this field is presented in [36], in which the inverted-pendulum theory is compared to the six determinants of gait theory. When using the inverted pendulum analogy to model human gait, the pendulum is formed by two main parts: (1) leg (including the thigh) of length *l* and mass $m_{l}$, and (2) upper part of the body with mass $m_{b}$ (this value of mass includes the other leg that will swing when walking).

Focusing on the legs, they can be approximated as uniform bars. Thus, the inertia with respect to *O* is given by [37,38]

(2) $$I_{l}=\frac{1}{3}m_{l}l^{2}$$

On the other hand, the upper part of the body is assumed to be a uniform cylinder. To determine its inertia, the Steiner Theorem can be applied [38]. Then,

(3) $$I_{b}=\frac{1}{12}m_{b}b^{2}+m_{b}{\left(\frac{b}{2}+l\right)}^{2}$$

Therefore, the total inertia of the human body when walking is

(4) $$I_{o}=I_{l}+I_{b}=\frac{1}{3}m_{l}l^{2}+\frac{1}{12}m_{b}b^{2}+m_{b}{\left(\frac{b}{2}+l\right)}^{2}$$

The human body has a proportion within its parts. For instance, the authors of [39] found that, for a male of 25.8 years of age, his height is around 8.58 times the height of his head. Another example is presented in [40], in which the authors set a relationship between the mass of the whole body and the mass of the limbs. Based on this criterion, let us call the relationship of human body height and mass as α and β, respectively. Then, Equation (4) can be rewritten in terms of the total human body mass *m* and height *h*, as shown in Equation (5).

(5) $$I_{o}=\frac{1}{3}\left(\beta_{l}m\right){\left(\alpha_{l}h\right)}^{2}+\frac{1}{12}\left(\beta_{b}m\right){\left(\alpha_{b}h\right)}^{2}+\left(\beta_{b}m\right){\left(\frac{\alpha_{b}h}{2}+\alpha_{l}h\right)}^{2}$$

By reducing terms,

(6) $$I_{o}=mh^{2}\left(\frac{1}{3}\beta_{l}\alpha_{l}^{2}+\frac{1}{12}\beta_{b}\alpha_{b}^{2}+\beta_{b}{\left(\alpha_{l}+\frac{\alpha_{b}}{2}\right)}^{2}\right)$$

### 3.3. Power Injected to Walk

**Figure 2:**
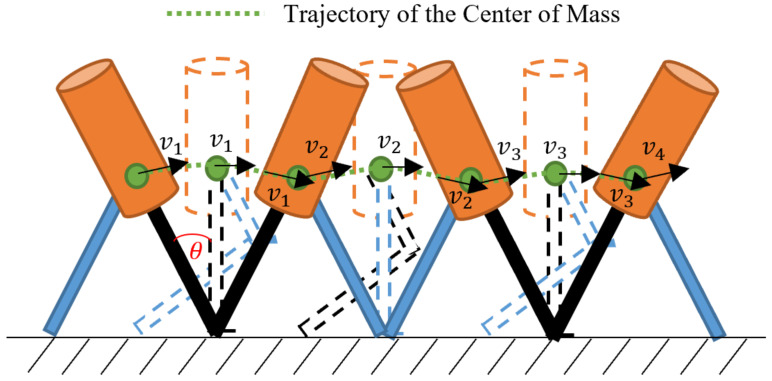
Motion of the centre of mass.

For this analysis, the air friction will be considered as negligible and it will be assumed that the centre of mass follows a trajectory as described in Figure 2.

The person moves due to the torque produced by the force he/she injects in each step *j*, that is,

(7) $$\tau_{0}=I_{o}\gamma \to F_{j}\times x=I_{o}\gamma \to |F_{j}||x|\sin\theta_{Fx}=I_{o}\gamma$$

where γ is the angular acceleration, which can be represented as a function of the tangential speed $v_{j}$, as follows,

(8) $$\gamma =v_{j}^{2}/x$$

On the other hand, the relationship between power $P_{j}$, force and speed is

(9) $$P_{j}=F_{j}\cdot v_{j}\to F_{j}=P_{j}\cdot v_{j}^{-1}$$

By replacing Equations (8) and (9) in Equation (7):

(10) $$|P_{j}\cdot v_{j}^{-1}||x|\sin\theta_{Fx}=I_{o}v_{j}^{2}/x$$

As *F* and *x* are orthogonal between them, the angle $\theta_{Fx}$ takes a value of 90${}^{\circ }$. Taking this fact into consideration, and solving *v* from (10), the result is as follows.

(11) $$|v_{j}|={\left(\frac{P_{j}{\left|x\right|}^{2}}{I_{o}}\right)}^{\frac{1}{3}}$$

The last formulation is valid under the following assumptions.

- When a person moves and has reached his/her limit speed (acceleration equal to zero), each leg is stiff during the time of its contact with the ground, like the spokes of a wheel, which thereby rotates without the benefit of a rim. Thus, each foot leaves contact with the ground at the same instant as the other one touches the ground. The flexing of the knee of the free leg serves only to keep that foot from contacting the ground as its leg swings forward, and such flexure does not consume significant energy or change the natural period of oscillation of the leg.
- The elasticity of the tendons and ligaments are ignored.
- The force on one foot acts at a fixed point.

### 3.4. Critical Human Walking Speed and Maximum Power Injected

With every step *j*, the person changes speed, and it depends on the power injected by the person. Nonetheless, there is a speed limit, attributed to the fact that walkers’ feet fall under gravity. Therefore, the acceleration cannot be greater than gravity.

For a body that experiments a circular motion, acceleration is defined by [37]

(12) $$\left|\gamma \right|=\frac{|v_{j}^{2}|}{x}$$

Solving Equation (12) for $v_{j}$, and considering a value of 9.8 $m/s^{2}$ for gravity, the maximum speed is as follows,

(13) $$|v_{max}|=\sqrt{9.8x}$$

By replacing Equation (13) in Equation (12) and solving for $P_{max}$,

(14) $$|P_{max}|=\frac{I_{o}\left({\sqrt{9.8x}}^{3}\right)}{x^{2}}$$

### 3.5. Human Walking Speed Behavior

**Figure 3:**
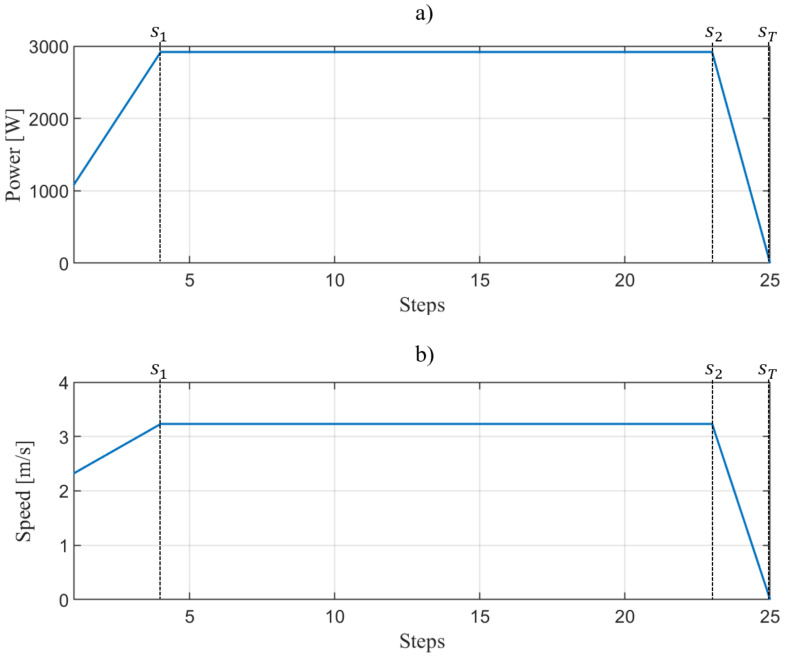
Human walking behaviour: (**a**) power and (**b**) speed [18,32].

Based on the analysis developed in [41], the human walking is described as follows, when the human walking starts, it requires an initial power, considered to be 1/3 of the maximum power. Then, in the next few steps, the person needs to inject more power, which starts increasing (for simplicity, this study assumes a linear increment) until the person reaches its normal speed ($v_{n}$) (assumed to be 90% of its maximum walking speed). Eventually, he/she will require to stop and, for that purpose, a fast decrease (approximated to be linear) in the injected power occurs until it becomes zero and, in that case, it is said that the person stops his/her motion. To better understand human speed behaviour, a schematic diagram of the power and speed of a man with BMI = OM, a total inertia of $I_{o}=113$ [kg/m${}^{2}$], x=1.143 m is presented in Figure 3.

In general, the mathematical formulation of speed is given by Equation (15).

(15) $$v=\left\{\begin{array}{cc} u_{1}s+w_{1} & \mathrm{for}\;0\leq s<s_{1}\\ v_{n} & \mathrm{for}\;s_{1}\leq s\leq s_{2}\\ u_{2}s & \mathrm{for}\;s_{2}<s\leq s_{3} \end{array}\right.$$

where

(16) $$w_{1}={\left(\frac{\frac{1}{3}P_{max}{\left|x\right|}^{2}}{I_{o}}\right)}^{\frac{1}{3}}/unit\;step\;in\;meters$$

(17) $$v_{n}={\left(\frac{0.90P_{max}{\left|x\right|}^{2}}{I_{o}}\right)}^{\frac{1}{3}}/unit\;step\;in\;meters$$

(18) $$u_{1}=\frac{v_{n}-w_{1}}{s_{1}}$$

(19) $$u_{2}=\frac{v_{n}}{s_{3}-s_{2}}$$

### 3.6. Distance with Every Step

In Figure 2, it is noteworthy that with one step a person travels a distance equal to

(20) $$unit\;step=s=2l\sin\theta =2\alpha_{l}h\sin\theta$$

The angle θ is typically about 25${}^{\circ }$ for human walking; therefore,

(21) $$s=0.845\alpha_{l}h$$

If a person walks a distance *D*, the total number of steps $s_{T}$ can be estimated using (22). Nevertheless, $s_{1}$ and $s_{2}$ varies depending on the sex and BMI of the person.

A sample of 25 people with different BMIs was used. The experiment consisted in making each individual walk. Then, the number of steps and time used to travel a certain distance were recorded. Table 1 was obtained based on the variance of the results. The values for $s_{1}$ and $s_{2}$ were obtained using the following formulations.

(22) $$s_{T}=D/s$$

### 3.7. Human Walking Time Calculation

If the human walking speed is known, then the time in which the person covers a distance *D* with $s_{T}$, can be determined. The analysis is carried out as follows.

The definition of speed in terms of steps is given below,

(23) $$v=\frac{ds}{dt}$$

then,

(24) $$t=\int_{0}^{s_{T}}v^{-1}ds$$

The speed equation is divided into three sections (as presented in Figure 3). Then, Equation (24) can be written as

(25) $$t=\int_{0}^{s_{1}}\frac{1}{u_{1}s+w_{1}}ds+\int_{s_{1}}^{s_{2}}\frac{1}{v_{n}}ds+\int_{s_{2}}^{s_{T}}\frac{1}{u_{2}s}ds$$

Solving the integrals:

(26) $$t={\left.\frac{\ln\left(u_{1}s+w_{1}\right)}{u_{1}}\right|}_{0}^{s_{1}}+{\left.\frac{s}{v_{n}}\right|}_{s_{1}}^{s_{2}}+{\left.\frac{\ln\left(s\right)}{u_{2}}\right|}_{s_{2}}^{s_{T}}$$

(27) $$t=\frac{\ln\left(u_{1}s_{1}+w_{1}\right)-\ln w_{1}}{u_{1}}+\frac{s_{2}-s_{1}}{v_{n}}+\frac{\ln s_{T}-\ln s_{2}}{u_{2}}$$

## 4. Simulation Environment

A relevant factor that affects the optimal evacuation route is the place in which the person is located when the natural hazard starts. This section presents the building structural features represented using graph theory. This representation is used to calculate the optimal evacuation route.

### 4.1. Building Design

The building’s design is based on a structural graph, where nodes represent a room, exit, ladder or another object. Moreover, relationships specify how nodes are connected to perform different analyses. The building is composed of a 30-story structure and a ground floor. Each floor is composed of several rooms, stairways and exits. The building’s area is 30 × 30 m${}^{2}$, with rooms of 5 × 10 m${}^{2}$ and 10 × 10 m${}^{2}$. The database used in the proposed model is Neo4j (Graph Database—GDB) [42]. The graph used to represent the building employs relationships that allow establishing links or union between objects, i.e., it shows the adjoining rooms and set outputs that connect the rooms, as well as the sequence of stories through a relationship (Up-stair or Down-stair). Each relationship in the proposed model is described below.

- A (BuildingNode) −>CONTAINS−>1..∗(FloorNode)
- A (FloorNode) −>CONTAINS−>1..∗(RoomNode) and/or 1..∗(StairNode) and/or 1..∗(ExitNode)
- A (StairNode) −>JOIN(UP−DOWN)−> Another (StairNode)
- A (RoomNode) −>JOIN(Left−Right−Behind−OnFront)−> Another (RoomNode)

Focusing on (RoomNode), (StairNode) and (ExitNode), they have the following information.

- X-start—It represents the x-position in the matrix from where the object starts.
- Y-start—It represents the y-position in the matrix from where the object starts.
- Height—It represents the length of the object, with reference to point (x-start, y-start).
- Width—It represents the width of the object, with reference to point (x-start, y-start).
- Soil—This attribute is only for room and floor; everything will depend on the type of surface within each object.

**Figure 4:**
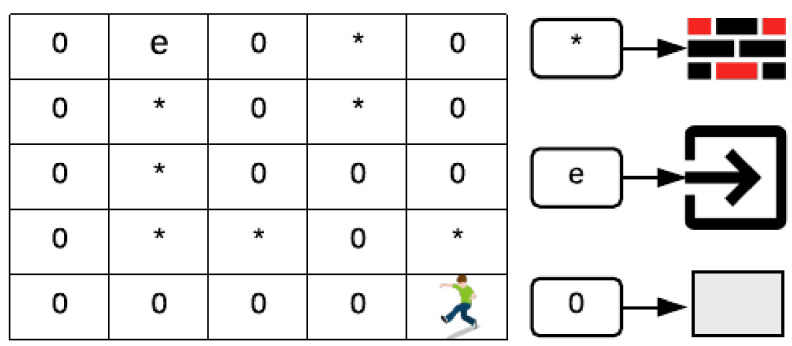
A symbolic representation of one floor inside the building.

Considering the previous information, the floor is designed with the following process. People are placed at a specific point (x,y) of any floor, as shown in Figure 4. Moreover, using (width,height) building attributes, different matrices are created with dimensions *M*x*N*(width,height). Depending on the configuration of the floor, a matrix is created per level with free boxes (0), locked (−2) and exits (−1); the exits can be of two types: room’s door or building exit. This will depend on the initial position of the person. Thus, we obtain a set of 31 matrices, one for the ground floor and the other 30 for each level of the building.

**Figure A1:**
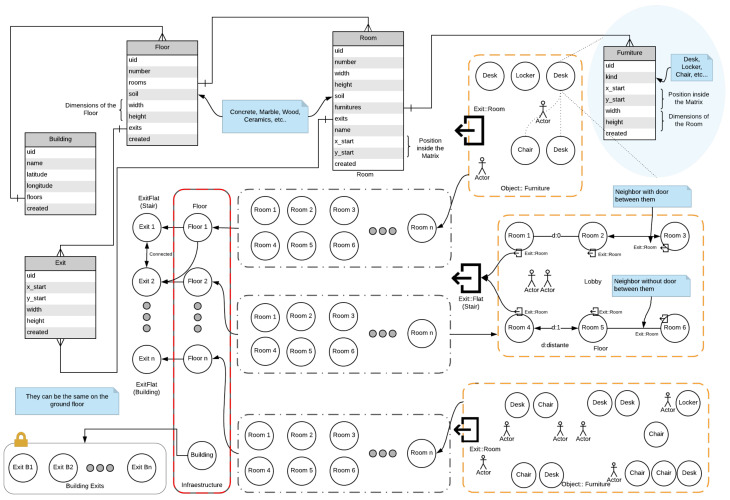
Model of the internal structure building, stored in a graph with their respective relationships and content nodes.

For more details, Figure A1 (Appendix A) shows the relational model graph of the building, how the nodes are connected to the relationships and the content of each node.

### 4.2. Walking Routes

To calculate the time that a person requires to get out of a building, three stages are defined as follows.

- *Graph*—A Web application (out of the scope of this paper) has been developed, where you can design a building (floors, stairs and rooms) to create a graph with this information.
- *Building*—This variable contains the design of the building, which is composed of arrays of arrays (Figure A1 (Appendix A)).
- *People inside the building*—This variable (Array[4232]) contains the attributes of the person, such as sex, weight, height, BMI and location inside the building.

The evacuation route is calculated for each person inside the building using the following as benchmarks: initial position of the person, and nearest floor exit, as shown in Figure 4. For every person, a twin matrix is created based on the floor in which he/she is located (one matrix per person). The characters used in the matrix free boxes (0), blocked (∗) and exits (e).

**Figure 5:**
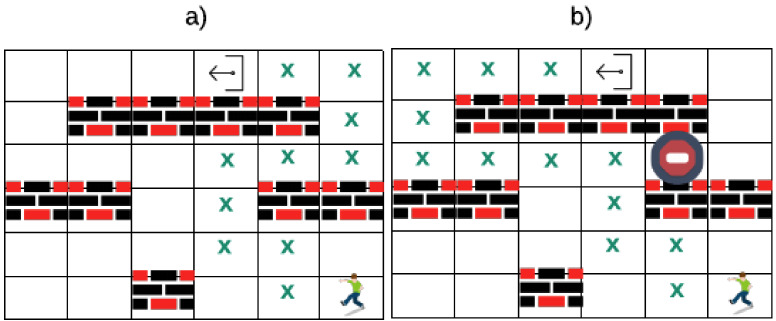
Evacuation route in a matrix using Breadth-First Search (BFS): (**a**) Non-blocked; (**b**) Blocked.

To find the evacuation optimal route, two algorithms are implemented. The former establishes the pathway to the nearest exit as a set (x,y) position. The latter determines the distance based on the position array. For instance, in Figure 5, a person is in position (6,6), and must be directed to the nearest exit in position (1,4). By using algorithm 2, the route is calculated, as shown in Figure 5a, with 11 boxes (distance) that the person has to walk. If the primary way is blocked, an alternative route is calculated, as shown in Figure 5b with 13 boxes to go. However, to calculate the evacuation route, algorithm 1 is used and each position (x,y) toward the nearest exit of the flat or the building is saved.

This procedure is then performed for each person (4232) inside the building, obtaining the optimal route from an initial position $\left(x_{i},y_{i}\right)$ to the closest exit $\left(x_{e},y_{e}\right)$. Then, the exit time is calculated by replacing the person’s variables (sex, height and weight) and the distance travelled inside the building using Equation (27).

### 4.3. Building Risk Index

**Figure 6:**
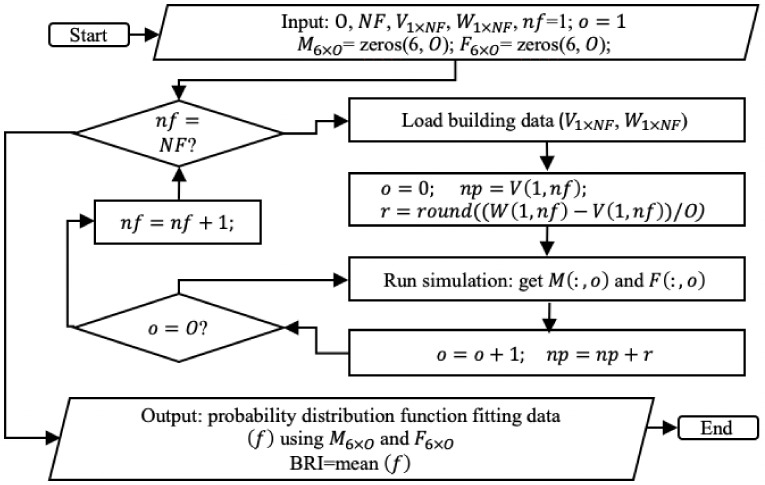
Flowchart of the process.

In this paper, the building risk index (BRI) refers to the statistical probability of not being capable of getting out of the building in the event of a natural hazard. In order to determine the BRI, a Monte Carlo simulation has been conducted. It starts by setting a random number of people with different features into different rooms and floors of the building. Then, a natural hazard condition is simulated and the optimal route for each person is determined by means of the algorithms previously described. As a result, the number of people who fail to get out of the building within the maximum evacuation time is obtained, and this value is saved. This process is repeated several times. With the saved data, the probability density function is obtained, and its mean represents the building risk index. Figure 6 presents a schematic diagram of the described process, where

- np: counter for the number of people inside the building
- nf: counter for the number of floors
- *o*: counter for the number of observations
- *O*: total number of observations
- *r*: increment of people in the building
- NF: total number of floors
- $V_{1xNF}$: vector that contains the minimum number of people that can be inside the building, which depends on the number of floors.
- $W_{1xNF}$: vector that contains the maximum number of people that can be inside the building, which depends on the number of floors.
- $M_{6x0}$: matrix that contains the percentage of men that did not survive. The columns represent the classification of BMI (UW, NO, OM, AO, SO, VSO), whereas the rows contain the observations.
- $F_{6x0}$: matrix that contains the percentage of women that did not survive. The columns represent the classification of BMI (UW, NO, OM, AO, SO, VSO), whereas the rows contain the observations.

## 5. Case Study

The experiment was carried out with a dataset containing a sample of 4232 people [43]; 2284 women and 1948 men, with different heights and weights. In addition, Table 2 shows the BMI classification established by the World Health Organization (WHO) [44], and the number of individuals for each BMI category in the experiment.

**Figure A2:**
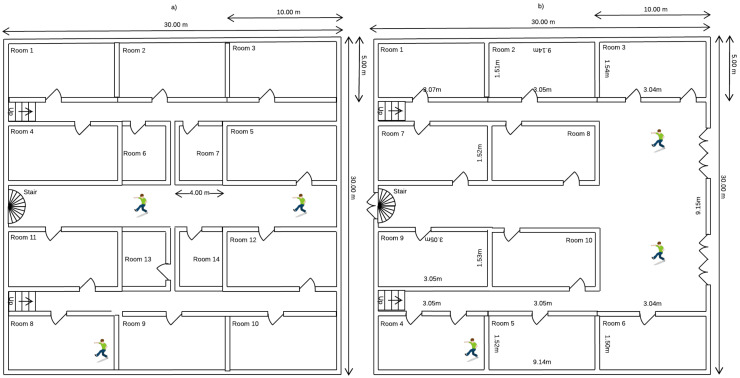
Scheme of the Building: (**a**) 30-storey building; (**b**) Ground Floor.

In addition, for the design of the building, the structure of the Institute of Applied Information Technology Building (InIT - ZHAW) was taken as a reference for the first floor, for the next floors the design is as shown in Figure A2.

## 6. Test Results

### 6.1. Exit Time Prediction

An example of the distribution of people for each flat, assuming a 30-storey building is as follows, [30, 138, 145, 147, 120, 144, 140, 140, 143, 132, 122, 142, 131, 154, 128, 144, 140, 145, 133, 145, 130, 152, 160, 145, 132, 140, 137, 155, 139, 141, 138]. This distribution is random for each sample of the experiment, which consists of 5000 samples. Every person in the experiment has a feature called field (x,y), which represents their position within their building floor. These values are used to obtain the results, which are classified according to BMI. In addition, there is a defined evacuation limit time (Ts) of 15 s and an additional increment of two seconds per floor. If the person leaves the building in a time lower than Ts, the person *“survives”*; otherwise, he/she is trapped inside the building. This is tagged as “1” or “0”, respectively, (it is used to determine the risk of the building).

The prediction of the exit time of a person is performed by using a Gaussian Process Regression (GPR (https://scikit-learn.org/stable/modules/gaussian_process.html)), due to its prediction accuracy compared to other models. A GPR is a supervised learning method designed to solve regression and probabilistic classification problems. In this case, it uses the kernel by default $\left(sigma_{0}=1.87e-05+noise_{level}=0.00951\right)$, obtaining a $R^{2}$ score of 0.90. To optimise this process, cross-validation is employed. It consists in adjusting a Gaussian process model using correlation parameters that are determined by maximum likelihood estimation (MLE). An anisotropic squared exponential correlation model with a constant regression model is assumed. Then, it computes a cross-validation estimate of the coefficient of determination ($R^{2}$), using the set of parameters found in the dataset (with K = 20-fold estimate of the coefficient of determination is $R^{2}=0.991$).

**Figure 7:**
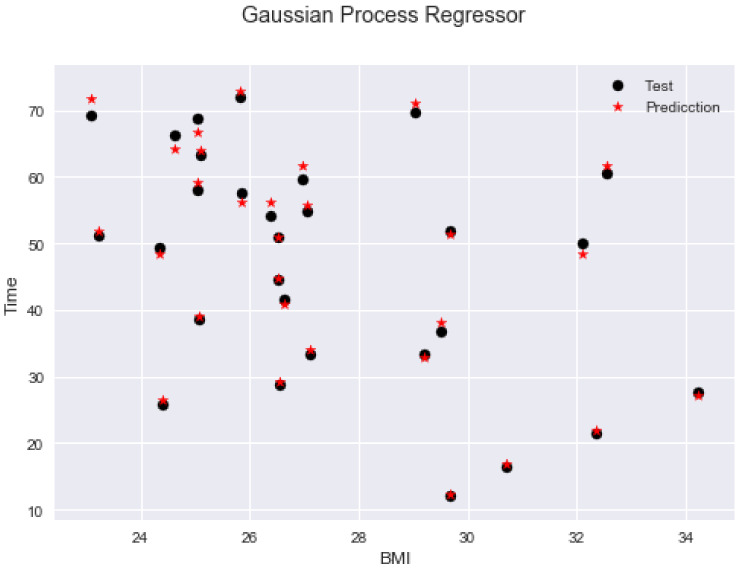
Predicting evacuation times with Gaussian Process Regression (GPR).

Figure 7 shows a sample of 30 people chosen at random to validate the GPR. In each of the cases, the time prediction shows a standard deviation ~0.09s, concerning the simulated time. In addition, to verify the proposed mathematical model presented in (27), Table 3 is presented. This table shows the evacuation time for people with different gender and BMI. The results are tabulated by group. The first group shows the evacuation time calculated using (27), whereas the second group shows the evacuation time based on statistical observation (simulation). The results reveal a relative error lower than 1% for all subject tests, which validates the mathematical model presented in (27).

### 6.2. Building Risk Index

**Figure 8:**
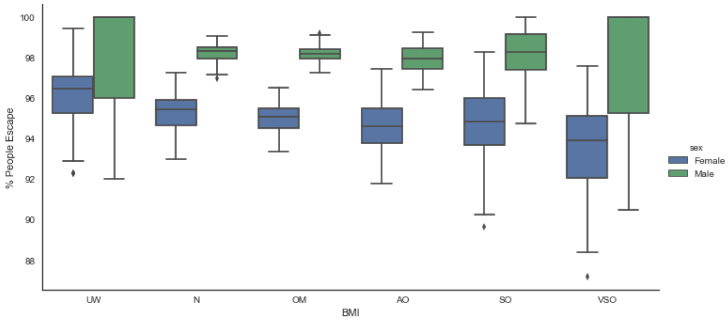
Evacuation times (People) classified by BMI.

Figure 8 shows a box plot of the percentage of people who escape the building within the evacuation time. These values are obtained by means of a Monte Carlo simulation, which is carried out with 5000 samples. It can be appreciated that, regardless of the BMI, males present a higher probability of evacuation than women. Regarding BMI, it presents an inverse relationship with the percentage of people who escape from the building. This last premise suggests that in order to reduce the risk, it is recommended to locate people inside the building by following a hierarchy from low to high, i.e., people with the highest BMI should be placed in the ground floor of the building and the ones with low BMI are suggested to be located in the upper floors.

**Figure 9:**
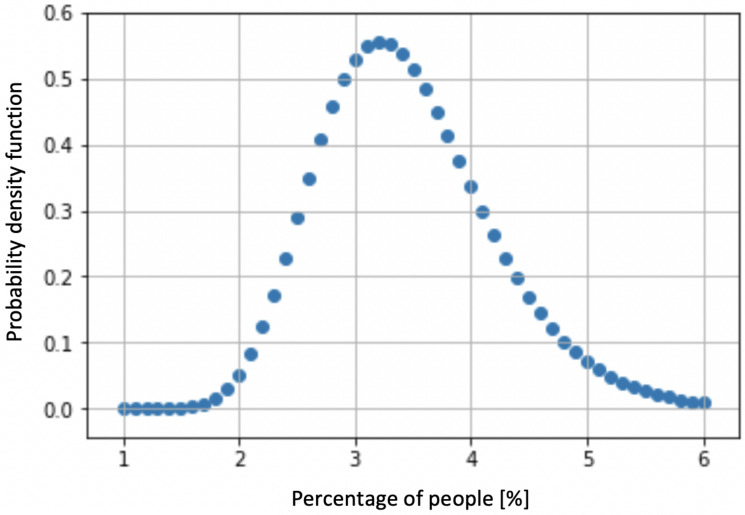
Birnbaum–Saunders probability function.

To evaluate the risk of the building, Figure 9 shows the probability density function of the people who did not evacuate the building. This graph represents the BRI and it follows a Birnbaum–Saunders probability function with a mean of 3.37. According to the Federal Emergency Management Agency (FEMA) (https://www.fema.gov/media-library-data/20130726-1730-25045-1580/femap_750.pdf), the mean BRI value categorizes the building as “safe” under earthquake conditions. This index provides useful information for safety managers, safety officers and safety analysts to carry out safety-based decision-making.

## 7. Discussion and Future Work

This paper proposes an innovative approach to show the impact of sex and BMI on human gait. The model incorporates graph theory to emulate building infrastructure and human physiology. The main engines of the approach are the Breadth-First Search algorithm to determine the optimal evacuation route, and the non-sequential Monte Carlo simulation to evaluate the risk of the building, in addition to the use given to the prediction of evacuation times to know future problems that may occur within a building.

The results show that BMI strongly affects the human gait, and the higher it becomes, the lower the probability of evacuating the building (considering a building with several floors). Moreover, sex presents an impact on human gait, that is, women exhibit a lower probability of evacuation than men.

The proposed approach presents a pathway to develop a more realistic model to enhance public safety. The formulation can be extended considering other metrics such as floors with slopes, leg positions at different angles (walking uphill, downstairs and so on). Besides the human physical fitness can significantly affect human gait. For instance, if an individual is healthy, he/she should have a higher probability of evacuation than those with a weak body or gait disorders. Last, the implementation of external forces that can destroy the roof and walls can be considered for future research.

## Figures and Tables

**Table 1:** Transition steps depending on the body mass index (BMI).

Sex	BMI	$s_{1}$	$s_{2}$
**Male**	Under Weight	∥rand(1,3)∥	∥rand(1,2)∥
	Normal	∥rand(2,3)∥	∥rand(1,3)∥
	Obesity Mild	∥rand(2,4)∥	∥rand(1,3)∥
	Average Obesity	∥rand(2,4)∥	∥rand(1,4)∥
	Severe Obesity	∥rand(2,5)∥	∥rand(2,5)∥
	Very Severe Obesity	∥rand(3,7)∥	∥rand(2,5)∥
**Female**	Under Weight	∥rand(2,3)∥	∥rand(1,2)∥
	Normal	∥rand(2,4)∥	∥rand(1,2)∥
	Obesity Mild	∥rand(3,5)∥	∥rand(1,3)∥
	Average Obesity	∥rand(3,6)∥	∥rand(1,4)∥
	Severe Obesity	∥rand(3,6)∥	∥rand(2,4)∥
	Very Severe Obesity	∥rand(3,6)∥	∥rand(2,4)∥

Note: the function rand(a,b) generates a uniformly random number between *a* and *b*, while the function round(a) round the value *x*.

**Table 2:** Classification of BMI per Individual.

BMI Male	BMI Women	Classification	Male	Female
<20	<20	UW	25	169
20–<25	20–<24	NO	531	656
25–<30	24–<29	OM	867	768
30–<35	29–<33	AO	390	353
35–<40	33–<37	SO	114	174
>=40	>=37	VSO	21	164

**Table 3:** Evacuation time verification.

Subject Test	Gender	BMI	Distance [m]	Evacuation Time [s]		Relative Error [%] $\mathrm{error}=|(t-t^{\prime })/t|x100$
				Using (27): *t*	Statistical Observation: $t^{\prime }$	
A	M	33.36	41	4.953312	4.986175	0.659082
B	M	26.54	121	12.32967	12.34569	0.129786
C	F	32.13	92	8.930232	8.950459	0.225988
D	M	26.62	59	6.056623	6.056424	0.003286
E	F	27.13	39	4.080984	4.082034	0.025722
F	M	26.62	80	8.265445	8.245791	0.238352
G	F	24.47	54	5.509876	5.511277	0.025421
H	F	25.38	66	6.952345	6.950091	0.032431
I	M	23.30	90	9.236540	9.234620	0.020791
J	M	27.45	21	2.213450	2.223788	0.464882

## Nomenclature

Some of the symbols and notations used throughout this manuscript are defined below for quick reference. Others are defined following their first appearances, as appropriated.

*x*	Centre of mass: position defined relative to an object or system of objects
*h*	Height of a person
*m*	Human body mass
*k*	Centre of mass sex factor: this is a constant defined by the sex of the person
*I*	Inertia: resistance, of any physical object, to any change in its velocity.
*O*	Pivot: reference point over which the torque is analysed
$I_{l}$	Inertia of the legs with respect to *O*
$m_{l}$	Mass of the legs
$I_{b}$	Inertia of the upper body (legs not included) with respect to *O*
$m_{b}$	Mass of the upper body (legs not included)
$I_{o}$	Inertia of the human body with respect to *O*
$\alpha_{l}$	Legs height rate: relationship between the length of the leg and human height
$\beta_{l}$	Legs mass rate: relationship between the mass of the legs and human body mass
$\alpha_{b}$	Upper body height rate: relationship between the length of the upper body and human height
$\beta_{b}$	Upper body mass rate: relationship between the mass of the upper body and human body mass
$\tau_{0}$	Torque that produces human body motion
*j*	Human body counter steps
γ	Human body angular acceleration
$v_{max}$	Maximum speed motion
$P_{max}$	Maximum power to get maximum speed motion
$P_{j}$	Power required to move the person in every step
$v_{j}$	Human body tangential speed with every step
*v*	Human body tangential speed per step
*s*	Distance travelled per step
$s_{T}$	Total distance travelled by the person
*t*	Time needed to travel the total distance

## Appendix A. Schemes & Algorithms

In this section, the building scheme and the algorithms for route calculation are shown.

**Algorithm 1:** Get the evacuation path
**Input**: grid, width, height, start **Output**: An array with each of the positions (x, y) where the person walks (path) wall, clear, goal = b’0’, b’*’, b’e’ queue = collections.deque([[start]]) seen = set([start]) **repeat**( path = queue.popleft() *x*, *y* = path[-1] **if** *grid[y][x] == goal* **then** ( return path **end** ( **repeat**( **if** *0 <= x2 < width and 0 <= y2 < height and grid[y2][x2] != wall and (x2, y2) not in see* **then** queue.append(path + [(x2, y2)]) seen.add((x2, y2)) **end**( **until** *x2, y2 in ((x+1,y), (x-1,y), (x,y+1), (x,y-1)*;( **until** queue;

**Algorithm 2:** Get the distance to walk with BFS
**Input**: room, width, height, $x_{person}$, $y_{person}$ **Output**: Exit’s x-position (row), exit’s y-position (col), distance the person must walk - # of boxes (distance), An array with each of the positions (x, y) where the person walks (path) source = QItem($y_{p}erson$ -1, $x_{p}erson$ -1,0); visited = np.zeros((int(height),int(width)), dtype=int) **repeat** **repeat** **if** *room[i][j] == b’0’* **then** visited[i][j] = 1 **else** visited[i][j] = 0 **end** **until** *range(0, int(width)*; **until** *range(0, int(height)*; *q* = queue.Queue(); *q*.put(source); visited[source.row][source.col] = 1 **repeat** *p* = *q*.get() **if** *room[p.row][p.col] == b’e’* **then** return p.row +1, p.col +1, dist, **bfsPathExit**(room, width, height, ($x_{person}$ -1,$y_{person}$ -1)) **end** The function *bfsPathExit* is calculated with the Algorithm 2. **if** *p.row - 1 >= 0 and visited[p.row - 1][p.col] == 0* **then** *q*.put(QItem(*p*.row - 1, *p*.col, *p*.dist + 1)) visited[*p*.row - 1][*p*.col] = 1 **end** **if** *p.row + 1 < int(height) and visited[p.row + 1][p.col] == 0* **then**( *q*.put(QItem(*p*.row + 1, *p*.col, *p*.dist + 1)) visited[*p*.row + 1][*p*.col] = 1 **end** **if** *p.col - 1 >= 0 and visited[p.row][p.col - 1] == 0* **then** *q*.put(QItem(*p*.row, *p*.col - 1, *p*.dist + 1)) visited[*p*.row][*p*.col - 1] = 1 **end** **if** *p.col + 1 < int(width) and visited[p.row][p.col + 1] == 0* **then** *q*.put(QItem(*p*.row,*p*.col + 1, *p*.dist + 1)) visited[*p*.row][*p*.col + 1] = 1 **end** **until** *q.empty() == False*; return -1
